# Genome sequencing, annotation and analysis of *Salmonella enterica* sub species *salamae* strain DMA-1

**DOI:** 10.1186/1757-4749-6-8

**Published:** 2014-04-11

**Authors:** Sathyaseelan Sathyabama, Gurwinder Kaur, Amit Arora, Sheenam Verma, Nida Mubin, Shanmugam Mayilraj, Javed N Agrewala

**Affiliations:** 1 Microbial Type Culture Collection and Gene bank (MTCC), CSIR-Institute of Microbial Technology, Sector 39-A, Chandigarh, India; 2 Immunology Laboratory, CSIR-Institute of Microbial Technology, Sector 39-A, Chandigarh, India

**Keywords:** *Salmonella enterica* subspecies *salamae*, RAST, EzTaxon

## Abstract

**Background:**

The genus *Salmonella* is Gram-negative which belongs to the family *Enterobacteriaceae*. In this study, we have sequenced the whole genome of the strain DMA-1, which was isolated from mouse stool sample and identified as *Salmonella enterica* subspecies *salamae.*

**Results:**

The strain DMA-1 was closely related at the 16S rRNA gene sequence level with the members of the genus *Salmonella*: *Salmonella enterica* subspecies *salamae* DSM 9220^T^ (100%), followed by *Salmonella enterica* subspecies *diarizonae* (99.1%), *Salmonella enterica* subspecies *enterica* (99.0%) and *Salmonella enterica* subspecies *indica* (98.5%). We obtained the draft genome of *S. enterica* subspecies *salamae* strain DMA-1 with a size of 4,826,209 bp and mean G+C content of 52.0 mol%.

**Conclusions:**

We for the first time, sequenced the entire genome of the strain DMA-1 which was isolated from the mouse stool sample and identified it as *Salmonella enterica,* sub species *salamae*. Further, we subjected the whole genome sequencing data for annotation that revealed several genes responsible for the pathogenesis, virulence, defense, metabolism and other genomic features.

## Background

During a study on identifying bacterial diversity of mouse stool samples, strain DMA-1 was isolated on tryptone soya agar (TSA, HiMedia, Mumbai, India). The strain DMA-1 was subjected to polyphasic taxonomic studies to identify the exact taxonomic status. The polyphasic taxonomical studies involved phenotypic, biochemical characterization and 16S rRNA gene sequencing that identified it as *Salmonella enteric*a subspecies *salamae*. The genus *Salmonella* was first proposed in 1952 by Kauffmann and Edwards [1]. Later on, it was emended in 1987 by Le Minor and Popoff [2]. The members of the genus *Salmonella* are Gram-negative, rod shaped, facultative anaerobes capable of aerobic respiration producing ATP and fermentation in the absence of oxygen. At present, the genus *Salmonella* consists of three species and six sub species. The *Salmonella enterica* subspecies include: *S. enterica* subspecies *enterica* (subspecies I), *S. enterica* subspecies *salamae* (subspecies II), *S. enterica* subspecies *arizonae* (subspecies IIIa), *S. enterica* subspecies *diarizonae* (subspecies IIIb), *S. enterica* subspecies *houtenae* (subspecies IV) and *S. enterica* subspecies *indica* (subspecies V). *S. enterica* includes the majority of *Salmonella* strains isolated from humans and warm blooded animals. But *Salmonella bongori* is typically obtained from cold blooded animals [3]. Most of the rodents present in laboratories are susceptible to *Salmonella* infections. Rodent feces cause infections via fecal-oral transmission, when a susceptible rodent ingests the bacteria present in their feed or contaminated drinking water. Immunocompromised caretakers of these rodents may get serious illness, which in some cases develop into life threatening diseases. Therefore in laboratories incoming rodents are quarantined and health monitoring for any rodent colony involves routine intestinal culture [4]. Infection caused by *Salmonella enterica* species is a major public health issue inflicting mortality globally. Contagious diseases caused by *S. enterica* depend mainly on their secreted proteins and adhesion from fimbrial and non-fimbrial sources, which produce biofilm and build contact with the host cell [5]. *S. enterica* also contains type I fimbriae, representative of other enteric bacteria, which help in attaching themselves to epithelial cells [6]. They are shorter in size than flagella but have a peritrichous distribution. Fimbriae are made up of major and minor protein subunits that make a cylinder around a hollow core [7]. Not all strains of *S. enterica* express fimbriae, but the fimbriaeted strains are more virulent [8].

*Salmonella* species could be differentiated on the basis of three antigens present on the cell: H antigen which is a flagellar antigen occurring only in one phase (1 or 2) and can interchange itself [9,10]; O antigen is a somatic antigen present on the outer membrane. The specificity of the O antigen is obtained by the character of the repeating units present on the outer O-polysaccharide chain [9]. The Vi antigen, virulence antigen which is a capsular polysaccharide that overlays the O antigen, but it is not possessed by all serovars. The capsule present in the *Salmonella* sp. is not essential for infection, yet it increases the infectivity by making it less visible to the body’s immune system [11].

*S. enterica* is also able to extract iron from host proteins by siderophores, which are formed by the cell when iron concentration is low, siderophores also contribute to virulence [12]. It also shows a mixed-acid heterofermentation of glucose to produce ATP. This process also produces CO_2_ and H_2_ with a variety of acid final -products, for example formate, acetate, lactate, and succinate [13]. *S. enterica* can nourish on maltose and maltodextrins with the help of type I ATP-binding cassette transporter. This system is present on a maltose regulon. In a maltose regulon the maltose enter into the cell by crossing the outer membrane through a homotrimeric maltoporin, after that it binds to a protein having pore-forming subunits that result in the translocation of an ATP-binding subunit across the inner-membrane where it is used by the cell [14]. *S. enterica* causes infectious diseases by entering the host cells that are present around the intestine, and attaching themselves to the cells of intestine, thereby causing ruffling response in the host cell membrane [5]. Ruffling is linked to a triggering response that results in macropinocytosis. The vesicles of macropinocytosis are large, and provide an efficient route for non-selective endocytosis of macromolecules [15]. The entry of the bacterial cells also harms the microvilli which are present on the cell surface, which causes disturbance in the white blood cells as they start to flood the mucosa thereby altering the homeostasis between absorption and secretion of the body [16].

*S. enterica* has a number of virulence factors like enterotoxin, endotoxin, and cytotoxin which are responsible for virulence. Enterotoxin causes diarrhea and vomiting in which the cells discharge huge amounts of fluid into the lumen. Endotoxin consists of toxic lipopolysaccharide which causes fever by inspiring host cells to discharge endogenous pyrogens. The cytotoxin interferes with protein synthesis in the host cell and leads to an efflux of calcium ions [15]. In some cases the infectious cells are transferred to the liver or spleen, where they multiply and return either to the host’s intestinal tract or get defecated [9].

Food contamination often occurs from infested feces that are present in polluted water, soil or other contaminated environments. *S. enterica* is responsible for causing many abdominal complications like gastroenteric disorders which involve stomach pain, cramps etc. When there is an imbalance between absorption and secretion, first signs include abdominal cramps, diarrhea, fever and nausea. Mostly the infection caused by food contamination cures within one week’s time, but more serious cases generally need fluoroquinolone or cephalosporin’s that eliminate *S. enterica* by interfering with their cell wall synthesis [17].

Presently only two species of the genus *Salmonella* have been sequenced for their whole genome: *Salmonella bongori* (two isolates), *Salmonella enterica*, forty three isolates including two isolates of *Salmonella enterica* subsp *salamae*, isolated from different sources, *Salmonella enteric*a subspecies *salamae* serovar 58:1, z13, z38:z6 strain 00-0163 [18] and *Salmonella enterica* subspecies *salamae* strain 3588/07 [Young *et al.* unpublished]. It is for the first time whole genome of strain DMA-1, isolated from mouse stool sample was sequenced by us. The whole genome sequencing was done in order to produce accurate reference genome which aids in identification and comparative genomic study. Moreover it also helps to analyze genes involved in virulence, disease and multidrug resistance.

## Methods

### Isolation, identification, DNA extraction, genome sequencing, assembly and annotation

*Salmonella enterica* subspecies *salamae* strain DMA-1 was isolated from mouse stool samples on 9^th^ October, 2012, and grown in tryptone soya agar (TSA, HiMedia, Mumbai, India). Genomic DNA extraction, amplification, 16S rRNA gene sequencing, phylogenetic analysis and G+C content were performed as described previously [19,20]. The genome of strain *Salmonella enterica,* subspecies *salamae* strain DMA-1 was sequenced at c-CAMP, Next Generation Genomic Facility, Bengaluru, India (http://www.ccamp.res.in), using a standard run of Illumina HiSeq 1000 sequencing technology. CLC Bio Workbench v6.0.2 (CLC Bio, Aarhus, Denmark) was employed for preprocessing of the data to remove the redundant and irrelevant information. A high quality 24,334,734 vector filtered reads at approximately 497.37 times coverage were used for final assembly (at word size of 45 and bubble size of 98). This draft genome comprising 4,826,209 bp was annotated with the help of RAST (Rapid Annotation using Subsystem Technology) system [21] server and RNAmmer 1.2 [22] server.

## Results and discussions

### Quality assurance: Identification

**Figure 1:**
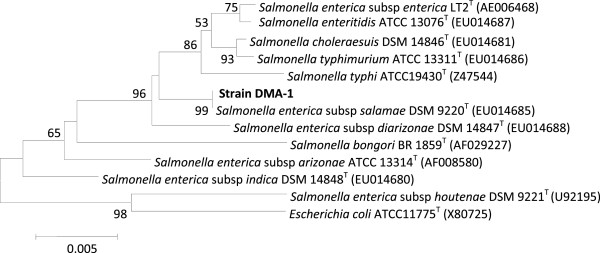
**Phylogenetic neighbour-joining tree based on 16S rRNA gene sequences showing the relationship between strain DMA-1 and related members of the genus *Salmonella.****Escherichia coli* (X80725) was used as an out-group. Bootstrap values (expressed as percentages of 100 replications) greater than 50% are given at nodes. Bar 0.005% sequence variation. GenBank accession numbers are given in parentheses.

Based on 16S rRNA gene sequence and phylogenetic analysis, strain DMA-1 was identified as *Salmonella enterica* subspecies *salamae*. Further polyphasic taxonomical data confirmed that the strain DMA-1 belongs to *Salmonella enterica* subspecies *salamae*. After two days of incubation at 30°C the colonies of the strain DMA-1 on TSA were cream colored and about 1-3 mm in diameter, circular, smooth, glistening, opaque, and convex with an entire margin. Cells were short-rod shaped, catalase-positive and oxidase-negative. Growth temperature ranged from 20°C and 37°C, optimum growth temperature was 30°C, no growth occurred at 42°C. pH range for growth was 5.2 – 7.5. NaCl was tolerated up to 2%. It was positive for hydrolysis of gelatin, production of hydrogen sulphide. It was negative for casein, starch hydrolysis, indole, urease, methyl red, Voges-Proskauer test and nitrate reduction. Acid was produced from D-glucose, D-dulcitol, and sorbitol but not from salicin and lactose. The G+C content of the genomic DNA was 52.0 mol%. A neighbour-joining tree (Figure 1) based on 16S rRNA gene sequence of the strain DMA-1 shows the phylogenetic relationship among the species of genus *Salmonella* and the organism formed a distinct branch along with *Salmonella enterica* subspecies *salamae* separated from the other members of the genus *Salmonella.*

### Initial Findings: Genomic features

**Figure 2:**
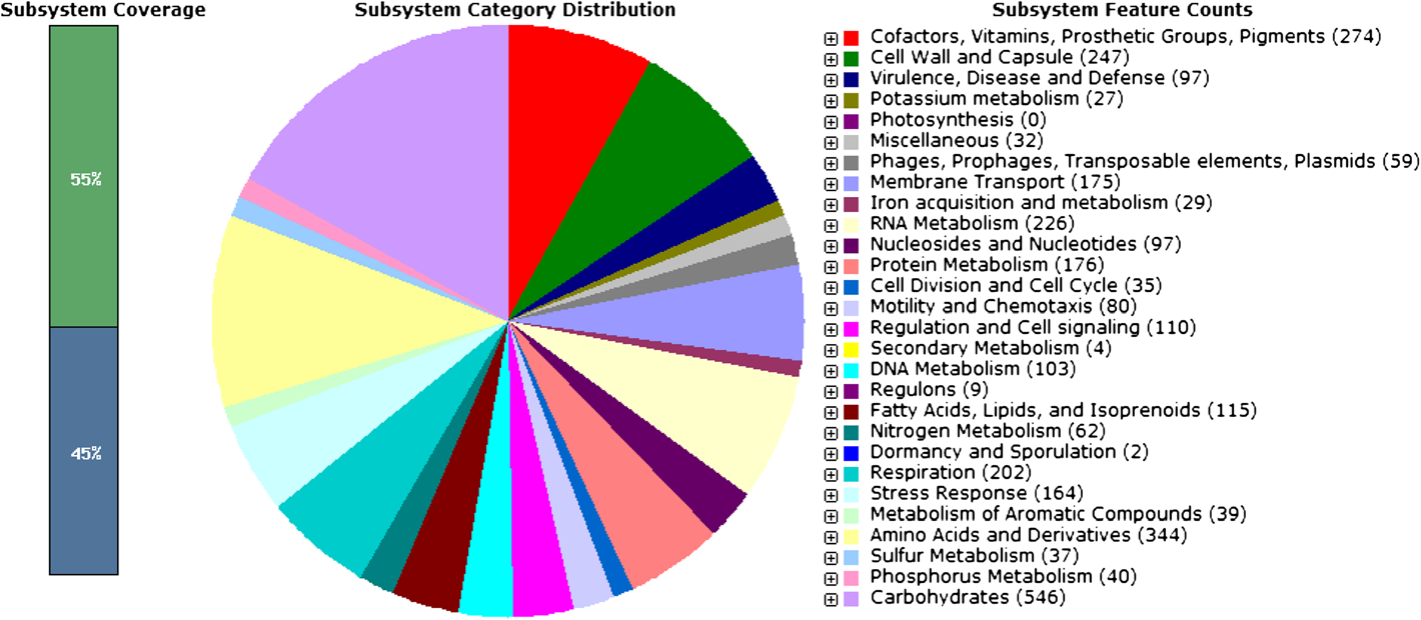
**Sub-system distribution of strain DMA-1 *Salmonella enterica *** **subspecies *salamae *** **(based on RAST annotation server).**

**Figure 3:**
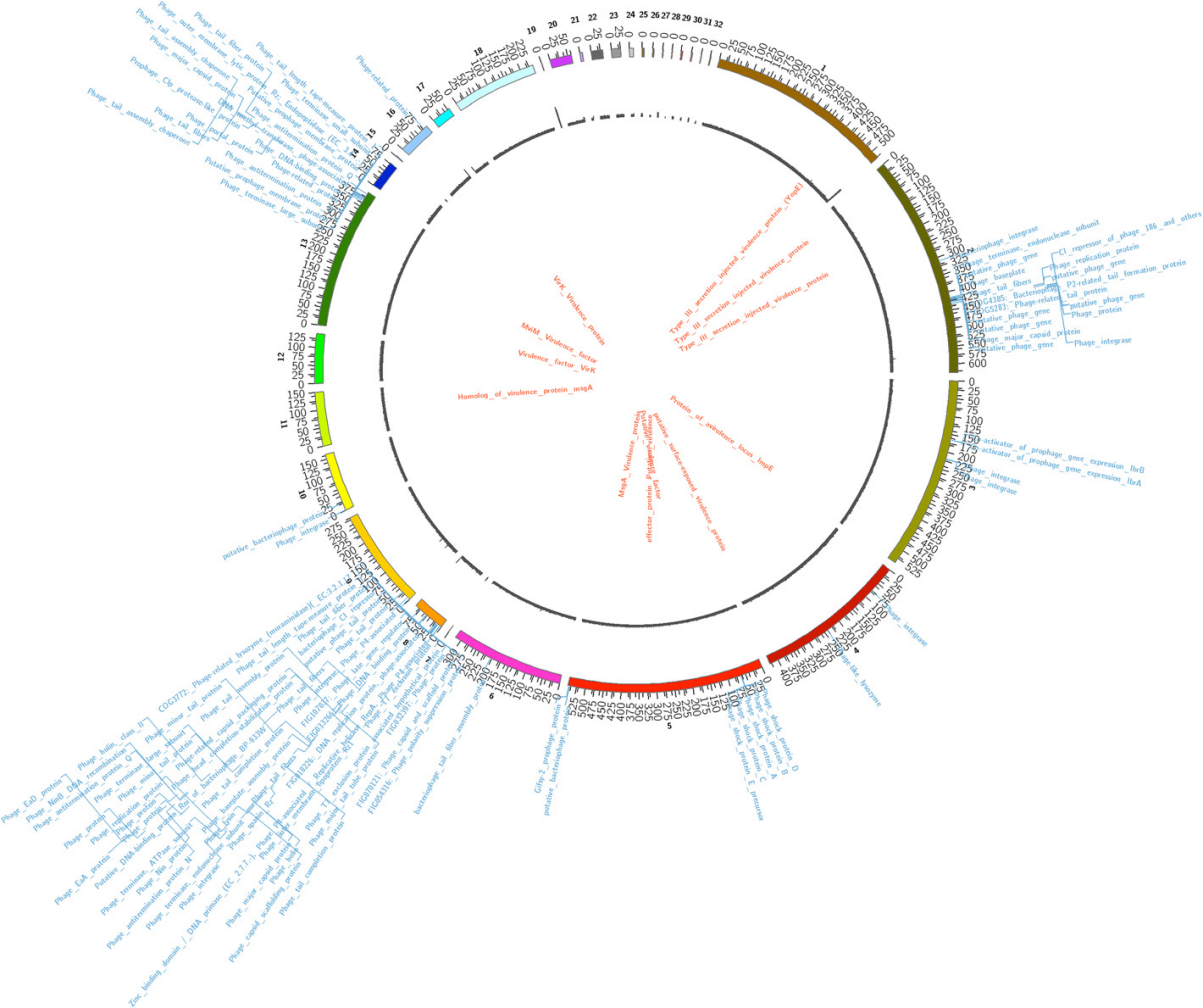
**Circular genome map of strain DMA-1 *Salmonella enterica *subspecies *salamae *showing the major genes and their regulators.** The 32 assembled contigs are shown by different coloured ideograms having their base-pair positions depicted at a scale of 1000 units. The coverage of the assembly at each base pair can be seen by the grey coloured track. Annotations descriptors for “virulent” genes [inner label: red] and “phage” related genes [outer label: blue] are mapped onto their respective contig positions. As many of the annotation descriptors occupy neighbouring positions on the contigs, the descriptors are stacked to allow better visualization.

The draft genome of *Salmonella enterica* subspecies *salamae* strain DMA-1 composed of 4,826,209 bp with G+C content of 52.0 mol%, 4,322 predicted CDSs and 69 RNAs. The final assembly contains 32 contigs with N_50_ contig length of 4,24,412 bp; the largest contig assembled measured 6,24,758 bp. RAST annotation indicates that *Salmonella enterica* subspecies *enterica* serovar *typhimurium* strain SL 1344 (score 519), *Salmonella enterica* subspecies *enterica* serovar *typhi* Ty2 (score 449), *Salmonella enterica* subspecies *enterica* serovar strain CVM 29188 (score 424) and *Salmonella enterica* (score 416) are the closest neighbors of the strain DMA-1. Summary of the basic characteristic features of the genomes is given in Table 1. The sub-system distribution of the strain DMA-1 is represented in Figure 2. Circular genome map of strain DMA-1 *Salmonella enterica* subspecies *salamae* showing the major genes and their regulators is shown as Figure 3.

**Table 1:** **Characteristic features of the annotated strain DMA-1 *Salmonella enterica, *** **sub species *salamae***

**Nucleotide**	**Count**	**Frequency (%)**	**G+C mol%**
Adenine (A)	1,155,424	23.9	
Cytosine (C)	1,265,442	26.2	52.0
Guanine (G)	1,246,212	25.8	
Thymine (T)	1,157,744	24.0	
Any nucleotide (N)	1,387	0.0	
**Characters**		**Length (bp)**	
N_75_		2,80,250	
N_50_		4,24,412	
N_25_		5,39,878	
Minimum		1,046	
Maximum		6,24,758	
Average		1,50,819	
Count		32	
Total		4,826,209	

### Genes involved in virulence, disease and defense, resistance to antibiotics and toxic compounds and multidrug resistance efflux pumps

Whole genome annotation at RAST server shows that *Salmonella enterica,* subspecies *salamae* strain DMA-1 contains genes related to the virulence, disease, defense and adhesion; mediator of hyperadherence YidE in *Enterobacteria* and its conserved region; 16 kDa heat shock protein A and B, outer membrane lipoprotein YidQ and HTH-type transcriptional regulator YidP. Bacteriocins, ribosomally synthesized antibacterial peptides; colicin V and bacteriocin production cluster; acetyl-coenzyme A carboxyl transferase beta chain (EC 6.4.1.2), amidophosphoribosyltransferase (EC 2.4.2.14), colicin V production protein, DedA protein, DedD protein, dihydrofolate synthase (EC 6.3.2.12), folylpolyglutamate synthase (EC 6.3.2.17) and tRNA pseudouridine synthase A (EC 4.2.1.70). Resistance to antibiotics and toxic compounds; the mdtABCD multidrug resistance cluster, multidrug transporter MdtC, response regulator BaeR, multidrug transporter MdtB, probable RND efflux membrane fusion protein, multidrug transporter MdtD and sensory histidine kinase BaeS. Multiple antibiotic resistance MAR loci; multiple antibiotic resistance protein MaeR, MaeA, MaeB and MaeC. Multidrug resistance efflux pumps; outer membrane lipoprotein NodT family, membrane fusion protein CmeA, outer membrane lipoprotein CmeC, multidrug and toxin extrusion (MATE) family efflux pump YdhE/NorM, macrolide export ATP-binding/permease protein MacB, MacA protein and type I secretion outer membrane protein, TolC precursor.

### Phages and prophages

The following proteins were identified in the genome of strain DMA-1: phage outer membrane lipoprotein Rz1, phage holin, class II, spanin Rz, lytic protein Rz, co-activator of prophage gene expression IbrA and IbrB, tail assembly protein, Nin protein, anti-termination protein Q, major capsid protein, capsid scaffolding protein and NinB DNA recombination.

### Functional based differential comparative genomic analysis

Functional based comparative genomic analysis for strains *Salmonella enterica* subspecies *salamae* DMA-1 and *Salmonella enterica* subspecies *enterica* SL483 were analyzed with the help of RAST (Rapid Annotation using Subsystem Technology) system. We identified a total of 3,437 genes (Additional file 1: Table S1) in which only 45 genes (Additional file 2: Table S2) were different between these two strains, based on their category, subcategory, subsystem and role. Some of the major differences include, the genes for category: virulence disease and defense having subcategory: adhesion, play role as 16 kDa heat shock protein B, mediator of hyperadherence YidE, outer membrane lipoprotein YidE, uncharacterized proteinYidR which are present in DMA-1 but absent in *S. enterica* subspecies *enterica*. Similar to this there are genes for category: phages, prophages, transposable elements, plasmids and having subcategory: phages, prophages having role as phage terminase small subunit, phage replication protein which are absent in *S. enterica* subspecies *enterica.*

## Conclusion

It was for the first time, whole genome sequencing of the strain DMA-1, isolated from mouse stool sample was carried out by us and the strain was identified as *Salmonella enterica* sub species *salamae.* Further, the genome sequencing data was subjected to annotation and the analysis revealed the genes responsible for the pathogenesis, virulence, defence, metabolism and other genomic features. The whole genome of *S. enterica* sub species *salamae* comprised of 4,826,209 bp in size with a G + C content of 52.0 mol%, having 4,322 predicted CDSs and 69 RNAs. The final assembly contained 32 contigs of total size 4,826,209 bp with N_50_ contig length of 4,24,412 bp; the largest contig assembled measured 6,24,758 bp.

### Future directions

There have been regular reports of *Salmonella enterica* subspecies *salamae* associated infections in Indian subcontinent. Therefore it becomes imperative to study the pathogenicity of the organism and its variants, which are very less explored and understood. The future work focuses on collecting various isolates of *Salmonella enterica* subsp *salamae* pertaining to different geographical locations across India, and to study their genome extensively. Whole genome sequencing of *Salmonella enterica* subspecies *salamae* would help in a deeper understanding of its pathogenicity, which enables us to identify new and improved drug targets for *Salmonella* associated diseases.

### Ethical clearance

The study was ethically approved by the Institutional Biosafety Committee (Ref/IBSC/2012-2/09) and Institutional Animal Ethics Committee (IAEC 13/01) of the CSIR-Institute of Microbial Technology, Chandigarh, India.

### Availability of supporting data

This Whole Genome Shotgun project has been deposited at DDBJ/EMBL/GenBank under the accession ATFA00000000. The version described in this paper is the first version, ATFA01000000.

## Competing interests

The authors have no competing interests.

## Authors’ contributions

Performed experiments: GK, SS, AA, SV and NM. Planned and executed experiments analyzed data and wrote manuscript: JNA and SM. All authors read and approved the final manuscript.

## Supplementary Material

Additional file 1: Table S1 Functional based differential comparative genomic analysis of (1) *Salmonella enterica* subspecies *salamae* strain DMA-1 and (2) *Salmonella enterica* subspecies *enterica* strain SL 483. Click here for file

Additional file 2: Table S2 Functional based (Virulence, Disease and Defense and Phages, Prophages, Transposable elements, Plasmids) differential comparative genomic analysis of (1) *Salmonella enterica* subspecies *salamae* strain DMA-1 and (2) *Salmonella enterica* subspecies *enterica* strain SL 483. Click here for file
