# Supplementary material for: Genome sequencing, annotation and analysis of Salmonella enterica sub species salamae strain DMA-1
Source: Gut Pathog. 2014 Apr 11;6:8. doi: 10.1186/1757-4749-6-8 (PMC4108123; doi:10.1186/1757-4749-6-8)
Supplement: Additional file 1: Table S1 — Functional based differential comparative genomic analysis of (1) Salmonella enterica subspecies salamae strain DMA-1 and (2) Salmonella enterica subspecies enterica strain SL 483. [file 1757-4749-6-8-S1.doc]

| **Category** | **Sub-category** | **Sub-system** | **Role** | **1** | **2** |
| --- | --- | --- | --- | --- | --- |
| Virulence, Disease and Defense | Adhesion | Mediator of hyperadherence YidE in Enterobacteria and its conserved region | 16 kDa heat shock protein B | yes | no |
| Virulence, Disease and Defense | Adhesion | Mediator of hyperadherence YidE in Enterobacteria and its conserved region | Mediator of hyperadherence YidE | yes | no |
| Virulence, Disease and Defense | Adhesion | Mediator of hyperadherence YidE in Enterobacteria and its conserved region | Outer membrane lipoprotein YidQ | yes | no |
| Virulence, Disease and Defense | Adhesion | Mediator of hyperadherence YidE in Enterobacteria and its conserved region | Uncharacterized protein YidR | yes | no |
| Virulence, Disease and Defense | Bacteriocins, ribosomally synthesized antibacterial peptides | Colicin V and Bacteriocin Production Cluster | Acetyl-coenzyme A carboxyl transferase beta chain (EC 6.4.1.2) | yes | yes |
| Virulence, Disease and Defense | Bacteriocins, ribosomally synthesized antibacterial peptides | Colicin V and Bacteriocin Production Cluster | Amidophosphoribosyltransferase (EC 2.4.2.14) | yes | yes |
| Virulence, Disease and Defense | Bacteriocins, ribosomally synthesized antibacterial peptides | Colicin V and Bacteriocin Production Cluster | Colicin V production protein | yes | yes |
| Virulence, Disease and Defense | Bacteriocins, ribosomally synthesized antibacterial peptides | Colicin V and Bacteriocin Production Cluster | DedA protein | yes | yes |
| Virulence, Disease and Defense | Bacteriocins, ribosomally synthesized antibacterial peptides | Colicin V and Bacteriocin Production Cluster | DedD protein | yes | yes |
| Virulence, Disease and Defense | Bacteriocins, ribosomally synthesized antibacterial peptides | Colicin V and Bacteriocin Production Cluster | Dihydrofolate synthase (EC 6.3.2.12) | yes | yes |
| Virulence, Disease and Defense | Bacteriocins, ribosomally synthesized antibacterial peptides | Colicin V and Bacteriocin Production Cluster | Folylpolyglutamate synthase (EC 6.3.2.17) | yes | yes |
| Virulence, Disease and Defense | Bacteriocins, ribosomally synthesized antibacterial peptides | Colicin V and Bacteriocin Production Cluster | tRNA pseudouridine synthase A (EC 4.2.1.70) | yes | yes |
| Virulence, Disease and Defense | Bacteriocins, ribosomally synthesized antibacterial peptides | Tolerance to colicin E2 | Conserved uncharacterized protein CreA | no | yes |
| Virulence, Disease and Defense | Bacteriocins, ribosomally synthesized antibacterial peptides | Tolerance to colicin E2 | Inner membrane protein CreD | no | yes |
| Virulence, Disease and Defense | Bacteriocins, ribosomally synthesized antibacterial peptides | Tolerance to colicin E2 | Two-component response regulator CreB | no | yes |
| Virulence, Disease and Defense | Bacteriocins, ribosomally synthesized antibacterial peptides | Tolerance to colicin E2 | Two-component response regulator CreC | no | yes |
| Virulence, Disease and Defense | Invasion and intracellular resistance | Mycobacterium virulence operon involved in DNA transcription | DNA-directed RNA polymerase beta subunit (EC 2.7.7.6) | yes | yes |
| Virulence, Disease and Defense | Invasion and intracellular resistance | Mycobacterium virulence operon involved in DNA transcription | DNA-directed RNA polymerase beta' subunit (EC 2.7.7.6) | yes | yes |
| Virulence, Disease and Defense | Invasion and intracellular resistance | Mycobacterium virulence operon involved in protein synthesis (LSU ribosomal proteins) | LSU ribosomal protein L20p | yes | yes |
| Virulence, Disease and Defense | Invasion and intracellular resistance | Mycobacterium virulence operon involved in protein synthesis (LSU ribosomal proteins) | LSU ribosomal protein L35p | yes | yes |
| Virulence, Disease and Defense | Invasion and intracellular resistance | Mycobacterium virulence operon involved in protein synthesis (LSU ribosomal proteins) | Translation initiation factor 3 | yes | yes |
| Virulence, Disease and Defense | Invasion and intracellular resistance | Listeria surface proteins: Internalin-like proteins | internalin, putative | yes | no |
| Virulence, Disease and Defense | Invasion and intracellular resistance | Mycobacterium virulence operon involved in protein synthesis (SSU ribosomal proteins) | Translation elongation factor Tu | no | yes |
| Virulence, Disease and Defense | Invasion and intracellular resistance | Salmonella invasion locus | Chaperone protein SicA (Salmonella invasin chaperone) | no | yes |
| Virulence, Disease and Defense | Invasion and intracellular resistance | Salmonella invasion locus | Surface presentation of antigens protein SpaQ | no | yes |
| Virulence, Disease and Defense | Resistance to antibiotics and toxic compounds | Adaptation to d-cysteine | Cystine ABC transporter, ATP-binding protein | yes | yes |
| Virulence, Disease and Defense | Resistance to antibiotics and toxic compounds | Adaptation to d-cysteine | Cystine ABC transporter, permease protein | yes | yes |
| Virulence, Disease and Defense | Resistance to antibiotics and toxic compounds | Adaptation to d-cysteine | D-cysteine desulfhydrase (EC 4.4.1.15) | yes | yes |
| Virulence, Disease and Defense | Resistance to antibiotics and toxic compounds | Aminoglycoside adenylyltransferases | Spectinomycin 9-O-adenylyltransferase | yes | yes |
| Virulence, Disease and Defense | Resistance to antibiotics and toxic compounds | Aminoglycoside adenylyltransferases | Streptomycin 3''-O-adenylyltransferase (EC 2.7.7.47) | yes | yes |
| Virulence, Disease and Defense | Resistance to antibiotics and toxic compounds | Arsenic resistance | Anion permease ArsB/NhaD-like | yes | yes |
| Virulence, Disease and Defense | Resistance to antibiotics and toxic compounds | Arsenic resistance | Arsenate reductase (EC 1.20.4.1) | yes | yes |
| Virulence, Disease and Defense | Resistance to antibiotics and toxic compounds | Arsenic resistance | Arsenical pump-driving ATPase (EC 3.6.3.16) | yes | yes |
| Virulence, Disease and Defense | Resistance to antibiotics and toxic compounds | Arsenic resistance | Arsenical resistance operon repressor | yes | yes |
| Virulence, Disease and Defense | Resistance to antibiotics and toxic compounds | Arsenic resistance | Arsenical resistance operon trans-acting repressor ArsD | yes | yes |
| Virulence, Disease and Defense | Resistance to antibiotics and toxic compounds | Arsenic resistance | Arsenical-resistance protein ACR3 | yes | yes |
| Virulence, Disease and Defense | Resistance to antibiotics and toxic compounds | Cobalt-zinc-cadmium resistance | Cation efflux system protein CusA | yes | yes |
| Virulence, Disease and Defense | Resistance to antibiotics and toxic compounds | Cobalt-zinc-cadmium resistance | Cobalt-zinc-cadmium resistance protein | yes | yes |
| Virulence, Disease and Defense | Resistance to antibiotics and toxic compounds | Cobalt-zinc-cadmium resistance | Cobalt-zinc-cadmium resistance protein CzcA | yes | yes |
| Virulence, Disease and Defense | Resistance to antibiotics and toxic compounds | Cobalt-zinc-cadmium resistance | Transcriptional regulator, MerR family | yes | yes |
| Virulence, Disease and Defense | Resistance to antibiotics and toxic compounds | Cobalt-zinc-cadmium resistance | Zinc transporter ZitB | yes | yes |
| Virulence, Disease and Defense | Resistance to antibiotics and toxic compounds | Copper homeostasis | Blue copper oxidase CueO precursor | yes | yes |
| Virulence, Disease and Defense | Resistance to antibiotics and toxic compounds | Copper homeostasis | Copper resistance protein D | yes | yes |
| Virulence, Disease and Defense | Resistance to antibiotics and toxic compounds | Copper homeostasis | Copper-translocating P-type ATPase (EC 3.6.3.4) | yes | yes |
| Virulence, Disease and Defense | Resistance to antibiotics and toxic compounds | Copper homeostasis | Cytochrome c heme lyase subunit CcmF | yes | yes |
| Virulence, Disease and Defense | Resistance to antibiotics and toxic compounds | Copper homeostasis | Cytochrome c heme lyase subunit CcmH | yes | yes |
| Virulence, Disease and Defense | Resistance to antibiotics and toxic compounds | Copper homeostasis: copper tolerance | Copper homeostasis protein CutE | yes | yes |
| Virulence, Disease and Defense | Resistance to antibiotics and toxic compounds | Copper homeostasis: copper tolerance | Copper homeostasis protein CutF precursor | yes | yes |
| Virulence, Disease and Defense | Resistance to antibiotics and toxic compounds | Copper homeostasis: copper tolerance | Cytoplasmic copper homeostasis protein CutC | yes | yes |
| Virulence, Disease and Defense | Resistance to antibiotics and toxic compounds | Copper homeostasis: copper tolerance | Magnesium and cobalt efflux protein CorC | yes | yes |
| Virulence, Disease and Defense | Resistance to antibiotics and toxic compounds | Copper homeostasis: copper tolerance | Membrane protein, suppressor for copper-sensitivity ScsB | yes | yes |
| Virulence, Disease and Defense | Resistance to antibiotics and toxic compounds | Copper homeostasis: copper tolerance | Membrane protein, suppressor for copper-sensitivity ScsD | yes | yes |
| Virulence, Disease and Defense | Resistance to antibiotics and toxic compounds | Copper homeostasis: copper tolerance | Secreted protein, suppressor for copper-sensitivity ScsC | yes | yes |
| Virulence, Disease and Defense | Resistance to antibiotics and toxic compounds | Copper homeostasis: copper tolerance | Suppression of copper sensitivity: putative copper binding protein ScsA | yes | yes |
| Virulence, Disease and Defense | Resistance to antibiotics and toxic compounds | Lysozyme inhibitors | Membrane-bound lysozyme inhibitor of c-type lysozyme | yes | yes |
| Virulence, Disease and Defense | Resistance to antibiotics and toxic compounds | Lysozyme inhibitors | Periplasmic lysozyme inhibitor of c-type lysozyme | yes | yes |
| Virulence, Disease and Defense | Resistance to antibiotics and toxic compounds | Mercuric reductase | Mercuric ion reductase (EC 1.16.1.1) | yes | yes |
| Virulence, Disease and Defense | Resistance to antibiotics and toxic compounds | Mercuric reductase | PF00070 family, FAD-dependent NAD(P)-disulphide oxidoreductase | yes | yes |
| Virulence, Disease and Defense | Resistance to antibiotics and toxic compounds | Mercury resistance operon | Mercuric ion reductase (EC 1.16.1.1) | yes | yes |
| Virulence, Disease and Defense | Resistance to antibiotics and toxic compounds | Multidrug Resistance Efflux Pumps | Macrolide export ATP-binding/permease protein MacB (EC 3.6.3.-) | yes | yes |
| Virulence, Disease and Defense | Resistance to antibiotics and toxic compounds | Multidrug Resistance Efflux Pumps | Macrolide-specific efflux protein MacA | yes | yes |
| Virulence, Disease and Defense | Resistance to antibiotics and toxic compounds | Multidrug Resistance Efflux Pumps | Multidrug and toxin extrusion (MATE) family efflux pump YdhE/NorM | yes | yes |
| Virulence, Disease and Defense | Resistance to antibiotics and toxic compounds | Multidrug Resistance Efflux Pumps | Multidrug-efflux transporter, major facilitator superfamily (MFS) (TC 2.A.1) | yes | yes |
| Virulence, Disease and Defense | Resistance to antibiotics and toxic compounds | Multidrug Resistance Efflux Pumps | RND efflux system, inner membrane transporter CmeB | yes | yes |
| Virulence, Disease and Defense | Resistance to antibiotics and toxic compounds | Multidrug Resistance Efflux Pumps | RND efflux system, membrane fusion protein CmeA | yes | yes |
| Virulence, Disease and Defense | Resistance to antibiotics and toxic compounds | Multidrug Resistance Efflux Pumps | RND efflux system, outer membrane lipoprotein CmeC | yes | yes |
| Virulence, Disease and Defense | Resistance to antibiotics and toxic compounds | Multidrug Resistance Efflux Pumps | RND efflux system, outer membrane lipoprotein, NodT family | yes | yes |
| Virulence, Disease and Defense | Resistance to antibiotics and toxic compounds | Multidrug Resistance Efflux Pumps | Transcription repressor of multidrug efflux pump acrAB operon, TetR (AcrR) family | yes | yes |
| Virulence, Disease and Defense | Resistance to antibiotics and toxic compounds | Multidrug Resistance Efflux Pumps | Type I secretion outer membrane protein, TolC precursor | yes | yes |
| Virulence, Disease and Defense | Resistance to antibiotics and toxic compounds | Multiple Antibiotic Resistance MAR locus | Multiple antibiotic resistance protein MarA | yes | yes |
| Virulence, Disease and Defense | Resistance to antibiotics and toxic compounds | Multiple Antibiotic Resistance MAR locus | Multiple antibiotic resistance protein MarB | yes | yes |
| Virulence, Disease and Defense | Resistance to antibiotics and toxic compounds | Multiple Antibiotic Resistance MAR locus | Multiple antibiotic resistance protein MarC | yes | yes |
| Virulence, Disease and Defense | Resistance to antibiotics and toxic compounds | Multiple Antibiotic Resistance MAR locus | Multiple antibiotic resistance protein MarR | yes | yes |
| Virulence, Disease and Defense | Resistance to antibiotics and toxic compounds | Resistance to fluoroquinolones | DNA gyrase subunit A (EC 5.99.1.3) | yes | yes |
| Virulence, Disease and Defense | Resistance to antibiotics and toxic compounds | Resistance to fluoroquinolones | DNA gyrase subunit B (EC 5.99.1.3) | yes | yes |
| Virulence, Disease and Defense | Resistance to antibiotics and toxic compounds | Resistance to fluoroquinolones | Topoisomerase IV subunit A (EC 5.99.1.-) | yes | yes |
| Virulence, Disease and Defense | Resistance to antibiotics and toxic compounds | Resistance to fluoroquinolones | Topoisomerase IV subunit B (EC 5.99.1.-) | yes | yes |
| Virulence, Disease and Defense | Resistance to antibiotics and toxic compounds | The mdtABCD multidrug resistance cluster | Multidrug transporter MdtB | yes | yes |
| Virulence, Disease and Defense | Resistance to antibiotics and toxic compounds | The mdtABCD multidrug resistance cluster | Multidrug transporter MdtC | yes | yes |
| Virulence, Disease and Defense | Resistance to antibiotics and toxic compounds | The mdtABCD multidrug resistance cluster | Multidrug transporter MdtD | yes | yes |
| Virulence, Disease and Defense | Resistance to antibiotics and toxic compounds | The mdtABCD multidrug resistance cluster | Probable RND efflux membrane fusion protein | yes | yes |
| Virulence, Disease and Defense | Resistance to antibiotics and toxic compounds | The mdtABCD multidrug resistance cluster | Response regulator BaeR | yes | yes |
| Virulence, Disease and Defense | Resistance to antibiotics and toxic compounds | The mdtABCD multidrug resistance cluster | Sensory histidine kinase BaeS | yes | yes |
| Virulence, Disease and Defense | Resistance to antibiotics and toxic compounds | Bile hydrolysis | Choloylglycine hydrolase (EC 3.5.1.24) | yes | no |
| Virulence, Disease and Defense | Resistance to antibiotics and toxic compounds | Copper homeostasis | Copper resistance protein C precursor | yes | no |
| Virulence, Disease and Defense | Resistance to antibiotics and toxic compounds | Adaptation to d-cysteine | L-Cystine ABC transporter, periplasmic cystine-binding protein | no | yes |
| Virulence, Disease and Defense | Resistance to antibiotics and toxic compounds | Fosfomycin resistance | Fosfomycin resistance protein FosA | no | yes |
| Virulence, Disease and Defense | Resistance to antibiotics and toxic compounds | Multidrug Resistance, Tripartite Systems Found in Gram Negative Bacteria | Membrane fusion component of tripartite multidrug resistance system | no | yes |
| Virulence, Disease and Defense | Resistance to antibiotics and toxic compounds | Multidrug Resistance, Tripartite Systems Found in Gram Negative Bacteria | Outer membrane component of tripartite multidrug resistance system | no | yes |
| Phages, Prophages, Transposable elements, Plasmids | Phages, Prophages | IbrA and IbrB: co-activators of prophage gene expression | Co-activator of prophage gene expression IbrA | yes | yes |
| Phages, Prophages, Transposable elements, Plasmids | Phages, Prophages | IbrA and IbrB: co-activators of prophage gene expression | Co-activator of prophage gene expression IbrB | yes | yes |
| Phages, Prophages, Transposable elements, Plasmids | Phages, Prophages | Phage capsid proteins | Phage head completion-stabilization protein | yes | yes |
| Phages, Prophages, Transposable elements, Plasmids | Phages, Prophages | Phage capsid proteins | Phage major capsid protein | yes | yes |
| Phages, Prophages, Transposable elements, Plasmids | Phages, Prophages | Phage DNA synthesis | DNA methyl transferase, phage-associated | yes | no |
| Phages, Prophages, Transposable elements, Plasmids | Phages, Prophages | Phage baseplate proteins | Phage baseplate | yes | no |
| Phages, Prophages, Transposable elements, Plasmids | Phages, Prophages | Phage capsid proteins | Phage capsid scaffolding protein | yes | no |
| Phages, Prophages, Transposable elements, Plasmids | Phages, Prophages | Phage lysis modules | Phage holin | yes | no |
| Phages, Prophages, Transposable elements, Plasmids | Phages, Prophages | Phage lysis modules | Phage holin, class II | yes | no |
| Phages, Prophages, Transposable elements, Plasmids | Phages, Prophages | Phage lysis modules | Phage lysin, 1,4-beta-N-acetylmuramidase (EC 3.2.1.17) or lysozyme | yes | no |
| Phages, Prophages, Transposable elements, Plasmids | Phages, Prophages | Phage lysis modules | Phage outer membrane lipoprotein Rz1 | yes | no |
| Phages, Prophages, Transposable elements, Plasmids | Phages, Prophages | Phage lysis modules | Phage outer membrane lytic protein Rz | yes | no |
| Phages, Prophages, Transposable elements, Plasmids | Phages, Prophages | Phage lysis modules | Phage spanin Rz | yes | no |
| Phages, Prophages, Transposable elements, Plasmids | Phages, Prophages | Phage nin genes - N-independent survival | Phage Nin protein | yes | no |
| Phages, Prophages, Transposable elements, Plasmids | Phages, Prophages | Phage nin genes - N-independent survival | Phage NinB DNA recombination | yes | no |
| Phages, Prophages, Transposable elements, Plasmids | Phages, Prophages | Phage nin genes - N-independent survival | Phage antitermination protein N | yes | no |
| Phages, Prophages, Transposable elements, Plasmids | Phages, Prophages | Phage nin genes - N-independent survival | Phage antitermination protein Q | yes | no |
| Phages, Prophages, Transposable elements, Plasmids | Phages, Prophages | Phage packaging machinery | Phage DNA-binding protein | yes | no |
| Phages, Prophages, Transposable elements, Plasmids | Phages, Prophages | Phage packaging machinery | Phage portal protein | yes | no |
| Phages, Prophages, Transposable elements, Plasmids | Phages, Prophages | Phage packaging machinery | Phage terminase large subunit | yes | no |
| Phages, Prophages, Transposable elements, Plasmids | Phages, Prophages | Phage packaging machinery | Phage terminase small subunit | yes | no |
| Phages, Prophages, Transposable elements, Plasmids | Phages, Prophages | Phage packaging machinery | Phage terminase, ATPase subunit | yes | no |
| Phages, Prophages, Transposable elements, Plasmids | Phages, Prophages | Phage replication | Phage replication protein | yes | no |
| Phages, Prophages, Transposable elements, Plasmids | Phages, Prophages | Phage tail fiber proteins | Phage tail fiber protein | yes | no |
| Phages, Prophages, Transposable elements, Plasmids | Phages, Prophages | Phage tail fiber proteins | Phage tail fibers | yes | no |
| Phages, Prophages, Transposable elements, Plasmids | Phages, Prophages | Phage tail proteins | Phage major tail tube protein | yes | no |
| Phages, Prophages, Transposable elements, Plasmids | Phages, Prophages | Phage tail proteins | Phage minor tail protein | yes | no |
| Phages, Prophages, Transposable elements, Plasmids | Phages, Prophages | Phage tail proteins | Phage tail assembly chaperone | yes | no |
| Phages, Prophages, Transposable elements, Plasmids | Phages, Prophages | Phage tail proteins | Phage tail assembly protein | yes | no |
| Phages, Prophages, Transposable elements, Plasmids | Phages, Prophages | Phage tail proteins | Phage tail length tape-measure protein 1 | yes | no |
| Phages, Prophages, Transposable elements, Plasmids | Phages, Prophages | Phage capsid proteins | Capsid scaffolding protein | no | yes |
| Iron acquisition and metabolism | Siderophores | Salmochelin-mediated Iron Acquisition | ABC transporter protein IroC | yes | yes |
| Iron acquisition and metabolism | Siderophores | Salmochelin-mediated Iron Acquisition | Glycosyltransferase IroB | yes | yes |
| Iron acquisition and metabolism | Siderophores | Salmochelin-mediated Iron Acquisition | Outer Membrane Siderophore Receptor IroN | yes | yes |
| Iron acquisition and metabolism | Siderophores | Salmochelin-mediated Iron Acquisition | Periplasmic esterase IroE | yes | yes |
| Iron acquisition and metabolism | Siderophores | Salmochelin-mediated Iron Acquisition | Trilactone hydrolase IroD | yes | yes |
| Iron acquisition and metabolism | Siderophores | Siderophore Aerobactin | Ferric hydroxamate ABC transporter (TC 3.A.1.14.3), ATP-binding protein FhuC | yes | yes |
| Iron acquisition and metabolism | Siderophores | Siderophore Aerobactin | Ferric hydroxamate ABC transporter (TC 3.A.1.14.3), periplasmic substrate binding protein FhuD | yes | yes |
| Iron acquisition and metabolism | Siderophores | Siderophore Aerobactin | Ferric hydroxamate ABC transporter (TC 3.A.1.14.3), permease component FhuB | yes | yes |
| Iron acquisition and metabolism | Siderophores | Siderophore Aerobactin | Ferric hydroxamate outer membrane receptor FhuA | yes | yes |
| Iron acquisition and metabolism | Siderophores | Siderophore Enterobactin | 2,3-dihydro-2,3-dihydroxybenzoate dehydrogenase (EC 1.3.1.28) [enterobactin] siderophore | yes | yes |
| Iron acquisition and metabolism | Siderophores | Siderophore Enterobactin | 2,3-dihydroxybenzoate-AMP ligase (EC 2.7.7.58) [enterobactin] siderophore | yes | yes |
| Iron acquisition and metabolism | Siderophores | Siderophore Enterobactin | 4'-phosphopantetheinyl transferase (EC 2.7.8.-) [enterobactin] siderophore | yes | yes |
| Iron acquisition and metabolism | Siderophores | Siderophore Enterobactin | Apo-aryl carrier domain of EntB | yes | yes |
| Iron acquisition and metabolism | Siderophores | Siderophore Enterobactin | Enterobactin esterase | yes | yes |
| Iron acquisition and metabolism | Siderophores | Siderophore Enterobactin | Enterobactin exporter EntS | yes | yes |
| Iron acquisition and metabolism | Siderophores | Siderophore Enterobactin | Enterobactin synthetase component F, serine activating enzyme (EC 2.7.7.-) | yes | yes |
| Iron acquisition and metabolism | Siderophores | Siderophore Enterobactin | FIG005032: Putative cytoplasmic protein YbdZ in enterobactin biosynthesis operon | yes | yes |
| Iron acquisition and metabolism | Siderophores | Siderophore Enterobactin | Ferric enterobactin transport ATP-binding protein FepC (TC 3.A.1.14.2) | yes | yes |
| Iron acquisition and metabolism | Siderophores | Siderophore Enterobactin | Ferric enterobactin transport system permease protein FepD (TC 3.A.1.14.2) | yes | yes |
| Iron acquisition and metabolism | Siderophores | Siderophore Enterobactin | Ferric enterobactin transport system permease protein FepG (TC 3.A.1.14.2) | yes | yes |
| Iron acquisition and metabolism | Siderophores | Siderophore Enterobactin | Ferric enterobactin uptake protein FepE | yes | yes |
| Iron acquisition and metabolism | Siderophores | Siderophore Enterobactin | Ferric enterobactin-binding periplasmic protein FepB (TC 3.A.1.14.2) | yes | yes |
| Iron acquisition and metabolism | Siderophores | Siderophore Enterobactin | Isochorismatase (EC 3.3.2.1) [enterobactin] siderophore | yes | yes |
| Iron acquisition and metabolism | Siderophores | Siderophore Enterobactin | Isochorismate synthase (EC 5.4.4.2) [enterobactin] siderophore | yes | yes |
| Iron acquisition and metabolism | Siderophores | Siderophore Enterobactin | Outer membrane receptor for ferric enterobactin and colicins B, D | yes | yes |
| Iron acquisition and metabolism | Siderophores | Siderophore Enterobactin | Proofreading thioesterase in enterobactin biosynthesis EntH | yes | yes |
