# Supplementary material for: Genome sequencing, annotation and analysis of Salmonella enterica sub species salamae strain DMA-1
Source: Gut Pathog. 2014 Apr 11;6:8. doi: 10.1186/1757-4749-6-8 (PMC4108123; doi:10.1186/1757-4749-6-8)
Supplement: Additional file 2: Table S2 — Functional based (Virulence, Disease and Defense and Phages, Prophages, Transposable elements, Plasmids) differential comparative genomic analysis of (1) Salmonella enterica subspecies salamae strain DMA-1 and (2) Salmonella enterica subspecies enterica strain SL 483. [file 1757-4749-6-8-S2.doc]

**Additional file 2. Table S2. Functional based (Virulence, Disease and Defense and Phages, Prophages, Transposable elements, Plasmids) differential comparative genomic analysis of (1) Salmonella enterica sub species salamae strain DMA-1 and (2) Salmonella enterica sub species enterica**strain SL 483.

| **Category** | **Sub-category** | **Sub-system** | **Role** | **1** | **2** |
| --- | --- | --- | --- | --- | --- |
| Virulence, Disease and Defense | Adhesion | Mediator of hyper adherence YidE in Enterobacteria and its conserved region | 16 kDa heat shock protein B | yes | no |
| Virulence, Disease and Defense | Adhesion | Mediator of hyper adherence YidE in Enterobacteria and its conserved region | Mediator of hyperadherence YidE | yes | no |
| Virulence, Disease and Defense | Adhesion | Mediator of hyper adherence YidE in Enterobacteria and its conserved region | Outer membrane lipoprotein YidQ | yes | no |
| Virulence, Disease and Defense | Adhesion | Mediator of hyper adherence YidE in Enterobacteria and its conserved region | Uncharacterized protein YidR | yes | no |
| Virulence, Disease and Defense | Bacteriocins, ribosomally synthesized antibacterial peptides | Tolerance to colicin E2 | Conserved uncharacterized protein CreA | no | yes |
| Virulence, Disease and Defense | Bacteriocins, ribosomally synthesized antibacterial peptides | Tolerance to colicin E2 | Inner membrane protein CreD | no | yes |
| Virulence, Disease and Defense | Bacteriocins, ribosomally synthesized antibacterial peptides | Tolerance to colicin E2 | Two-component response regulator CreB | no | yes |
| Virulence, Disease and Defense | Bacteriocins, ribosomally synthesized antibacterial peptides | Tolerance to colicin E2 | Two-component response regulator CreC | no | yes |
| Virulence, Disease and Defense | Invasion and intracellular resistance | Listeria surface proteins: Internalin-like proteins | Internalin, putative | yes | no |
| Virulence, Disease and Defense | Invasion and intracellular resistance | Mycobacterium virulence operon involved in protein synthesis (SSU ribosomal proteins) | Translation elongation factor Tu | no | yes |
| Virulence, Disease and Defense | Invasion and intracellular resistance | Salmonella invasion locus | Chaperone protein SicA (Salmonella invasin chaperone) | no | yes |
| Virulence, Disease and Defense | Invasion and intracellular resistance | Salmonella invasion locus | Surface presentation of antigens protein SpaQ | no | yes |
| Virulence, Disease and Defense | Resistance to antibiotics and toxic compounds | Bile hydrolysis | Choloylglycine hydrolase (EC 3.5.1.24) | yes | no |
| Virulence, Disease and Defense | Resistance to antibiotics and toxic compounds | Copper homeostasis | Copper resistance protein C precursor | yes | no |
| Virulence, Disease and Defense | Resistance to antibiotics and toxic compounds | Adaptation to d-cysteine | L-Cystine ABC transporter, periplasmic cystine-binding protein | no | yes |
| Virulence, Disease and Defense | Resistance to antibiotics and toxic compounds | Fosfomycin resistance | Fosfomycin resistance protein FosA | no | yes |
| Virulence, Disease and Defense | Resistance to antibiotics and toxic compounds | Multidrug Resistance, Tripartite Systems Found in Gram Negative Bacteria | Membrane fusion component of tripartite multidrug resistance system | no | yes |
| Virulence, Disease and Defense | Resistance to antibiotics and toxic compounds | Multidrug Resistance, Tripartite Systems Found in Gram Negative Bacteria | Outer membrane component of tripartite multidrug resistance system | no | yes |
| Phages, Prophages, Transposable elements, Plasmids | Phages, Prophages | Phage DNA synthesis | DNA methyl transferase, phage-associated | yes | no |
| Phages, Prophages, Transposable elements, Plasmids | Phages, Prophages | Phage baseplate proteins | Phage baseplate | yes | no |
| Phages, Prophages, Transposable elements, Plasmids | Phages, Prophages | Phage capsid proteins | Phage capsid scaffolding protein | yes | no |
| Phages, Prophages, Transposable elements, Plasmids | Phages, Prophages | Phage lysis modules | Phage holin | yes | no |
| Phages, Prophages, Transposable elements, Plasmids | Phages, Prophages | Phage lysis modules | Phage holin, class II | yes | no |
| Phages, Prophages, Transposable elements, Plasmids | Phages, Prophages | Phage lysis modules | Phage lysin, 1,4-beta-N-acetylmuramidase (EC 3.2.1.17) or lysozyme | yes | no |
| Phages, Prophages, Transposable elements, Plasmids | Phages, Prophages | Phage lysis modules | Phage outer membrane lipoprotein Rz1 | yes | no |
| Phages, Prophages, Transposable elements, Plasmids | Phages, Prophages | Phage lysis modules | Phage outer membrane lytic protein Rz | yes | no |
| Phages, Prophages, Transposable elements, Plasmids | Phages, Prophages | Phage lysis modules | Phage spanin Rz | yes | no |
| Phages, Prophages, Transposable elements, Plasmids | Phages, Prophages | Phage nin genes - N-independent survival | Phage Nin protein | yes | no |
| Phages, Prophages, Transposable elements, Plasmids | Phages, Prophages | Phage nin genes - N-independent survival | Phage NinB DNA recombination | yes | no |
| Phages, Prophages, Transposable elements, Plasmids | Phages, Prophages | Phage nin genes - N-independent survival | Phage antitermination protein N | yes | no |
| Phages, Prophages, Transposable elements, Plasmids | Phages, Prophages | Phage nin genes - N-independent survival | Phage antitermination protein Q | yes | no |
| Phages, Prophages, Transposable elements, Plasmids | Phages, Prophages | Phage packaging machinery | Phage DNA-binding protein | yes | no |
| Phages, Prophages, Transposable elements, Plasmids | Phages, Prophages | Phage packaging machinery | Phage portal protein | yes | no |
| Phages, Prophages, Transposable elements, Plasmids | Phages, Prophages | Phage packaging machinery | Phage terminase large subunit | yes | no |
| Phages, Prophages, Transposable elements, Plasmids | Phages, Prophages | Phage packaging machinery | Phage terminase small subunit | yes | no |
| Phages, Prophages, Transposable elements, Plasmids | Phages, Prophages | Phage packaging machinery | Phage terminase, ATPase subunit | yes | no |
| Phages, Prophages, Transposable elements, Plasmids | Phages, Prophages | Phage replication | Phage replication protein | yes | no |
| Phages, Prophages, Transposable elements, Plasmids | Phages, Prophages | Phage tail fiber proteins | Phage tail fiber protein | yes | no |
| Phages, Prophages, Transposable elements, Plasmids | Phages, Prophages | Phage tail fiber proteins | Phage tail fibers | yes | no |
| Phages, Prophages, Transposable elements, Plasmids | Phages, Prophages | Phage tail proteins | Phage major tail tube protein | yes | no |
| Phages, Prophages, Transposable elements, Plasmids | Phages, Prophages | Phage tail proteins | Phage minor tail protein | yes | no |
| Phages, Prophages, Transposable elements, Plasmids | Phages, Prophages | Phage tail proteins | Phage tail assembly chaperone | yes | no |
| Phages, Prophages, Transposable elements, Plasmids | Phages, Prophages | Phage tail proteins | Phage tail assembly protein | yes | no |
| Phages, Prophages, Transposable elements, Plasmids | Phages, Prophages | Phage tail proteins | Phage tail length tape-measure protein 1 | yes | no |
| Phages, Prophages, Transposable elements, Plasmids | Phages, Prophages | Phage capsid proteins | Capsid scaffolding protein | no | yes |
